# Hepatocyte‐like cells generated by direct reprogramming from murine somatic cells can repopulate decellularized livers

**DOI:** 10.1002/bit.26784

**Published:** 2018-09-17

**Authors:** Chen Chen, Iris Pla‐Palacín, Pedro M. Baptista, Peng Shang, Loes A. Oosterhoff, Monique E. van Wolferen, Louis C. Penning, Niels Geijsen, Bart Spee

**Affiliations:** ^1^ Department of Clinical Sciences of Companion Animals Faculty of Veterinary Medicine, Utrecht University Utrecht The Netherlands; ^2^ Hubrecht Institute‐KNAW and University Medical Centre Utrecht Utrecht The Netherlands; ^3^ Instituto de Investigación Sanitaria de Aragón (IIS Aragón) Zaragoza Spain; ^4^ Centro de Investigación Biomédica en Red en el Área Temática de Enfermedades Hepáticas (CIBERehd) Madrid Spain; ^5^ Fundación ARAID Zaragoza Spain; ^6^ Instituto de Investigación Sanitaria de la Fundación Jiménez Díaz Madrid Spain; ^7^ Department of Biomedical and Aerospace Engineering Universidad Carlos III de Madrid Madrid Spain

**Keywords:** decellularized liver tissue, direct reprogramming, induced hepatocytes (iHeps), polycistronic expression

## Abstract

Direct reprogramming represents an easy technique to generate induced hepatocytes (iHeps) from somatic cells. However, current protocols are accompanied by several drawbacks as iHeps are heterogenous and lack fully mature phenotypes of primary hepatocytes. Here, we established a polycistronic expression system to induce the direct reprogramming of mouse embryonic fibroblasts towards hepatocytes. The resulting iHeps are homogenous and display key properties of primary hepatocytes, such as expression of hepatocyte markers, albumin secretion, and presence of liver transaminases. iHeps also possess the capacity to repopulate decellularized liver tissue and exhibit enhanced hepatic maturation. As such, we present a novel strategy to generate homogenous and functional iHeps for applications in tissue engineering and cell therapy.

## 1 INTRODUCTION

Currently, orthotopic liver transplantation is the only cure for severe acute and chronic liver diseases. Unfortunately, the shortage of donor livers is a growing problem. In Europe alone, 22% of the patients die while on the waiting list for a liver transplant (Brangel & Undine, 2016). Therefore, the development of new therapeutic strategies such as stem cell transplantation is of utmost importance. Although several types of stem cells show the potential to be differentiated into hepatocyte‐like cells, the procurement of the stem cells as well as differentiation protocols is inefficient, incomplete, and still far from application in human medicine (Schwartz, Fleming, Khetani, & Bhatia, 2014; Wu & Tao, 2012).

One promising alternative for generating hepatocytes is the direct reprogramming technique. As from the first direct reprogramming experiments inducing myoblasts over 30 years ago, more and more types of tissue have been generated with this technique (Kelaini, Cochrane, & Margariti, 2014). Transcription factors together with other epigenetic modifiers coordinately play an important role in maintaining cellular identities by regulating cell‐type specific gene expression programs. Based on this theory, direct reprogramming was aimed at the forced expression of these key transcription factors to activate the regulatory network supporting a specific cell fate. During direct reprogramming, one somatic cell (e.g., fibroblast) is transdifferentiated into another somatic cell (e.g., hepatocyte) without intermediate stages of pluripotency. Due to this feature, direct reprogramming represents a more reproducible and time‐efficient technique compared with pluripotent stem cell‐based differentiation. Direct reprogramming has been shown to allow the generation of induced hepatocytes (iHeps) from many types of somatic cells (Du et al., 2014; Huang et al., 2011; Sekiya & Suzuki, 2011). These iHeps acquired hepatocyte function to some extent and could extend the survival of mouse models with lethal liver disease after cell transplantation. However, full maturation of these cells in vitro has thus far not been achieved.

In the liver, hepatocytes are surrounded by extracellular matrix (ECM), of which the dominant ECM components are collagens (type I, III, IV, and V). Natural‐based ECM, especially type IV collagen, has been routinely used for maintaining hepatocytes and iHeps (Huang et al., 2011; LeCluyse, Bullock, & Parkinson, 1996). Apart from serving as the scaffold for hepatocytes, ECM also performs signaling function by storage and release of numerous growth factors, hormones, enzymes, and cytokines. Considering the complexity of natural ECM, a single or combination of several types of collagens may not be able to mimic the genuine in vivo microenvironment for hepatocytes. Therefore, the decellularized liver tissue may be a better alternative. Previously, we and others have reported that decellularized liver tissue could provide an excellent environment for the in vitro differentiation of hepatic stem cells (Vyas et al., 2018; Wang et al., 2011) as well as maintenance of primary hepatocytes (Soto‐Gutierrez et al., 2011). However, it is still unknown whether iHeps can repopulate decellularized liver tissue and whether the maturation of iHeps can be enhanced by this more natural environment.

In this study, we report the efficient generation of iHeps from mouse embryonic fibroblasts (MEFs) using a polycistronic system expressing transcription factors including Forkhead Box A3 (*Foxa3*), HNF1 Homeobox A (*Hnf1α*), and GATA Binding Protein 4 (*Gata4*). Different from previously used combinations of separate vectors, each encoding one or two genes, the all‐in‐one polycistronic expression system induces homogenous iHep population as well as fluorescent marker. These iHeps morphologically and functionally resemble primary hepatocytes. Furthermore, we demonstrate that iHeps can repopulate decellularized liver tissue. More important, iHeps cultured with decellularized liver tissue exhibit high level of maturation. This iHep‐on‐decellularized‐liver‐tissue system may serve as an optimal model for drug activity and toxicity screening.

## 2 MATERIAL AND METHODS

### 2.1 Assembly of polycistronic lentiviral vectors

To construct the lentiviral hepatocyte‐generating vector, we used a third‐generation lentiviral vector (pRRL‐PPT‐SFFV‐OKSM‐EGFP, kindly provided by A. Schambach, Hannover, Germany; Warlich et al., 2011). The region encoding Oct4, Klf4, Sox9, and Myc (OKSM) was removed from the original vector by *Bam*HI (New England Biolabs, Ipswich, MA) digestion. The three modules encoding FOXA3‐T2A, HNF1A‐E2A, and GATA4 were amplified by polymerase chain reaction with monocistronic vectors separately expressing *Foxa3*, *Hnf1a,* and *Gata4* (FHG) as templates (kindly provided by L. Hui, Shanghai, China; Huang et al., 2011). Each of these modules contained 15‐bp overlaps at their ends. Ligation of all the modules was based on the In‐Fusion HD Cloning system (Clontech Laboratories Inc, Mountain View, CA), in which the In‐Fusion enzyme can fuse all the modules and the backbone by recognizing 15‐bp overlaps at their ends.

### 2.2 Cell culture, lentivirus production, and lentiviral transduction

The human embryonic kidney line (HEK) 293T and mouse embryonic fibroblasts were cultured in Dulbecco's modiﬁed Eagle's medium (DMEM, Gibco, Dublin, Ireland) supplemented with 10% v/v fetal bovine serum (FBS, Gibco) and 1% v/v penicillin/streptomycin. Primary hepatocytes were isolated by a two‐step perfusion procedure from C57BL/6 mice (Severgnini et al., 2012). Viable hepatocytes were isolated by Percoll medium according to the manufacturer's instructions (GE Healthcare, Chicago, IL).

Virus production was performed as previously described (Nantasanti et al., 2015). In short, 7 × 10^6^ HEK‐293T cells were plated 24 hr before transfection (Day 1) in 15 cm dishes. On Day 0, cells were transfected by linear polyethylenimine (PEI; Polysciences Inc, Warrington, PA, 1 μg DNA: 5 μg PEI) with 45 μg lentiviral vector, 3.6 μg HDM‐Hgpm2, 3.6 μg RC‐CMV‐Rev1b, 3.6 μg HDM‐tat1b, and 7.2 μg HDM‐VSV‐G. Media was refreshed after 12–16 hr. Supernatant containing virus was harvested on Days 2–4. On Day 4, filtered supernatant was centrifuged at 72,000 *g* for 2 hr at 4°C. The pellet was resuspended in 150 μl sterile phosphate‐buffered saline (PBS) with 1% w/v bovine serum albumin, and aliquots were stored at −80°C until use.

### 2.3 Generation of iHeps

For viral transduction, on Day 0, MEFs were incubated with concentrated lentivirus (1:10,000) containing the reprogramming factors and 8 μg/ml of Polybrene (Sigma‐Aldrich, St. Louis, MO). On Day 1, MEFs were washed by HBSS (Gibco) twice and cultured with fresh MEF medium. On Day 2, iHep medium, containing a 1:1 mixture of DMEM/F‐12, supplemented with 10% FBS, 20 ng/ml hepatocyte growth factor, 20 ng/ml epidermal growth factor, 1 μg/ml insulin, 10^−7^ M dexamethasone, 10 mM nicotinamide, 2 mM l‐glutamine, 50 μM β‐mercaptoethanol, and 1% v/v penicillin/streptomycin. Cells were surpassed on Days 7 and 14. The culture dishes were precoated with type I collagen (Millipore, Burlington, MA). Medium was refreshed every 2 days.

### 2.4 RNA isolation, complementary DNA synthesis, and RT‐qPCR

RNA was isolated from MEFs, primary hepatocytes, and two dimension‐cultured iHeps using the RNeasy Micro Kit according to the manufacturer's instructions (Qiagen, Hilden, Germany). For isolating RNA from the iHeps on the disks, we first used PBS to wash the disks, and then added lysis buffer (RLT buffer, from RNeasy micro kit, Qiagen) directly. After 5 min of incubation, all the lysates were collected for further procedures. Complementary DNA (cDNA) was obtained using the iScript™ cDNA synthesis kit as described by the manufacturer (Bio‐Rad, Hercules, CA). Relative gene expression of the selected genes was measured using RT‐qPCR. Primer design, validation, RT‐qPCR conditions, and data analysis were performed as previously described (van Steenbeek et al., 2013). Normalization was performed using the reference gene hypoxanthine phosphoribosyl transferase. Details of primers are listed in Table S1.

### 2.5 Immunofluorescence analysis

Cells were fixed in 4% paraformaldehyde for 60 min and permeabilized with PBS containing 0.3% v/v Triton X‐100 for 30 min. Cells cultured on decellularized liver disks (DLD) were incubated with 0.01 g/ml NaBH_4_ solution to reduce the background generated by the liver disks. Primary antibodies were incubated overnight. Secondary antibodies were incubated at room temperature for 2 hr. Tissues were then incubated with 5 µM Alexa Fluor^®^ 488 (Molecular Probes, Eugene, OR) or Alexa Fluor^®^ 647 (for cells cultured on DLD) according to the manufacturer's instructions. Nuclei were stained with DAPI (Sigma‐Aldrich). Tissues were mounted on slides with ProLong^®^ Diamond Antifade Mounting Medium (Molecular Probes). Images were acquired using the Leica SPE‐II confocal system. Antibody details for each protein are shown in Table S2.

### 2.6 Immunoblot analysis

For primary mouse hepatocytes and transduced and untransduced MEFs, whole cell lysates were prepared using radioimmunoprecipitation assay (RIPA) lysis buffer containing 50 mM Tris HCl, 150 mM NaCl, 1% v/v NP‐40, 0.25% w/v sodium deoxycholate, 1 mM ethylenediaminetetraacetic acid, 1 mM sodium fluoride, 1 mM sodium orthovanadate, 1 µg/ml aprotinin, and 1 mM phenylmethylsulfonyl fluoride (PMSF) (Sigma‐Aldrich). Protein concentration was measured by the DC Protein Assay (Bio‐Rad). Fifty micrograms of total protein for each sample was loaded in a 10% SDS‐PAGE gel, transferred to a 0.45 μm nitrocellulose membrane (Bio‐Rad), and blocked with the ECL Blocking agent (GE Healthcare). The blots were probed with anti‐FOXA3 (Thermo Fisher Scientific, Waltham, MA, PA1‐813, dilution 1:500), anti‐HNF1A (Thermo Fisher Scientific, PA5‐35356, dilution 1:1,000), and anti‐GATA4 (Abcam, Cambridge, UK, ab84593, dilution 1:1,000) overnight at 4°C and subsequently incubated with HRP‐conjugated anti‐rabbit (R&D systems, Minneapolis, MI, HAF008, dilution 1:5,000) for 1 hr at room temperature. Luminescence induced by the Amersham ECL western blot analysis Detection Reagent (GE Healthcare) was measured with a ChemiDoc XRS Imager (Bio‐Rad).

### 2.7 Hepatocyte functional tests

For measurement of albumin (*Alb*) secretion, iHeps, MEFs, and primary hepatocytes were cultured in serum‐free medium. After 24 hr, culture medium was collected. Protein in the medium was concentrated using Amicon Ultra centrifugal filters (Millipore), and the amount of *Alb* was measured using a DxC‐600 Beckman chemistry analyzer (Beckman Coulter, Brea, CA). The values were normalized for total cell number.

For the measurement of cytochrome P450 activity, iHeps, MEFs, and primary hepatocytes were incubated in 50 μM luciferin‐PFBE substrate (Promega, Madison, WI) in culture medium (Gibco) containing 10% FBS for 8 hr at 37°C. Cyp3a activity was then measured with a luminometer using the P450‐Glo cytochrome P450 assay kit according to the manufacturer's instructions (Promega).

For the measurement of the expression of hepatic enzymes in two dimension‐cultured iHeps, cells were lysed in distilled water. The alkaline phosphatase (ALP), gamma‐glutamyl transferase (GGT), lactate dehydrogenase (LDH), glutamate dehydrogenase (GLDH), and aspartate aminotransferase (AST) in the lysate were measured using the DxC‐600 Beckman chemistry analyzer (Beckman Coulter), and values were normalized to total cell number. For the measurement of the expression of hepatic enzymes in DLD‐iHeps, two disks of cells were pooled together to generate enough material for each liver enzyme test as described above; the results of those tests were normalized to the value of the alamarBlue test (Thermo Fisher Scientific), which indicates the number of viable cells.

### 2.8 Decellularized liver disk preparation

Briefly, livers from 4‐ to 8‐month‐old cadaveric rats were harvested with intact vessels (the animals were previously used by other researchers of our institution and killed on completion of their institutional animal care and use committee (IACUC) approved experiments). The livers were then cannulated through the portal vein and successively perfused at the rate of 1 ml/min with 0.5 L distilled water, 5 L detergent comprising 1% v/v Triton‐X 100 (Sigma‐Aldrich) and 0.1% v/v ammonium hydroxide (Sigma‐Aldrich), and 10 L distilled water to wash out the decellularization detergent. Decellularized livers with a level of DNA removal higher than 90% were obtained with this method.

To obtain liver disks, decellularized livers were cut into wedges and embedded in plastic molds (Sakura Finetek, Europe, Alphen a/d Rijn, the Netherlands) with OCT and frozen at −80°C. These cryopreserved liver wedges were mounted onto a cryomicrotome (CM 1950, Leica Biosystems, Nussloch, Germany) to obtain liver ECM sections of 250 μm thickness. A 4‐mm‐diameter biopsy punch was used to generate a disk from the liver sections, and the disks were placed in 96‐well plates. After multiple washes with PBS, the disks were sterilized by UV irradiation for 2 hr. Disks were stored in PBS at 4°C until use.

### 2.9 Recellularization of iHeps on DLD

The seeding procedure was performed as previously described (Vyas et al., 2018). Briefly, 2.5 × 10^5^ iHeps were suspended in 10 μl iHep medium for each disk. Disks were placed in 96‐well round bottom microwell plates (Nunc, Roskilde, Denmark). The cell suspension was slowly pipetted on top of each disk and incubated for about an hour at 37°C to allow the cells to attach. Afterwards, 200 μl additional iHep medium was added to each well. As a two‐dimensional control, the same number of iHeps were seeded on collagen (type I) coated 96‐well tissue culture plates.

### 2.10 Statistical analysis

Statistical analysis and graphs were performed using GraphPad Prism 7.0 (GraphPad Software Inc., San Diego, CA). See the figure legends for details on specific statistical tests run and *p* values calculated for each experiment.

## 3 RESULTS

### 3.1 Design of lentiviral vectors inducing hepatic reprogramming gene expression in mouse embryonic fibroblasts

**Figure 1:**
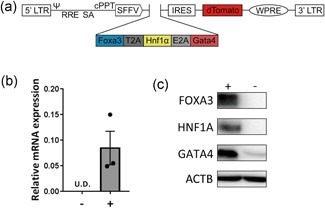
Design and efficacy of the polycistronic lentiviral vector. (a) Modular configuration of the self‐inactivating (SIN) vector backbones for expression of the hepatocyte transcription factors *Foxa3*, *Hnf1α*, and *Gata4*. (b) Relative mRNA expression of the exogenous genes in MEFs without transduction (–) and with transduction of FHG lentivirus after 5 days (+). Data are shown as mean ± *SEM* of three independent experiments for each group. (c) Protein expression of FOXA3, HNF1A, GATA4, and ACTB in untransduced (–) and transduced (+) MEFs. ψ: packaging signal; cPPT: central polypurine tract; FHG: Foxa3, Hnf1α and Gata4; Foxa3: Forkhead Box A3; Gata4: GATA Binding Protein 4; Hnf1α: HNF1 Homeobox A; IRES: internal ribosomal entry site; MEF: mouse embryonic fibroblasts; LTR: long terminal repeat; RRE: rev‐responsive element; SA: splice acceptor; *SEM*: standard error of the mean; SFFV: spleen focus‐forming virus U3 promoter; UD: undetectable; WPRE: woodchuck hepatitis virus [Color figure can be viewed at wileyonlinelibrary.com]

Previously, Warlich et al. (2011) developed a modular lentiviral vector system for generating human and mouse pluripotent stem cells (iPSCs). Apart from the region encoding the iPSC reprogramming factors (OKSM), the main modules in this vector are spleen focus‐forming virus U3 promoter (SFFV), which has been proven to mediate efficient expression in fibroblasts (Baum, Hegewisch‐Becker, Eckert, Stocking, & Ostertag, 1995); an internal ribosome entry site (IRES)‐driven fluorescence marker, dTomato, for imaging/cell tracking studies; a posttranscriptional regulatory element derived from the woodchuck hepatitis virus to enhance lentiviral vector titer and expression (Schambach et al., 2006); as well as the necessary elements for lentivirus production (Figure 1a). Hepatocyte‐generating factors FHG (Huang et al., 2011) were amplified independently and inserted in the vector to replace OKSM. FHG were separated by thosea asigna virus 2A (T2A) and equine rhinitis A virus 2A (E2A) sequences, which have previously been shown to mediate complete separation of recombinant proteins (Kim et al., 2011). MEFs were transduced with FHG lentivirus and RNA and protein samples were collected after 5 days. Gene expression analysis, using a qPCR primer set amplifying the region on *Hnf1α* and E2A, confirmed the exogenous expression of FHG (Figure 1b). Immunoblot analysis further showed that FHG were highly expressed in transduced MEFs, whereas they were not or very low expressed in untransduced MEFs (Figure 1c).

### 3.2 Generation of mouse iHeps with the polycistronic expression system

**Figure 2:**
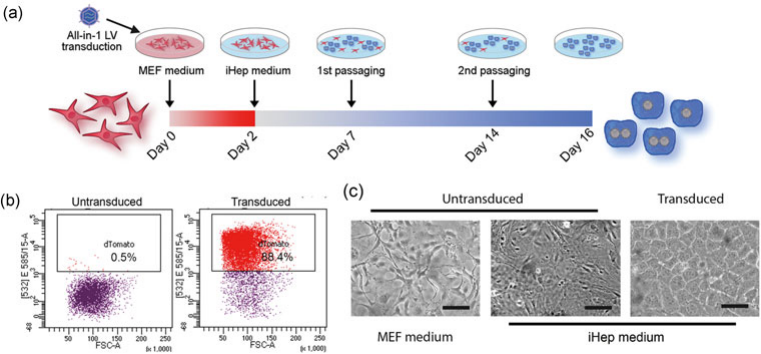
Generation of iHeps from MEFs and morphology. (a) Schematic showing the generation of iHeps. Cells were cultured in MEF medium for 2 days after transduction. From Day 2, cells were cultured in iHep medium. Cells were subcultured twice around Day 7 and Day 14, when reaching confluency. (b) Representative flow cytometry analysis of dTomato expression in untransduced MEFs (left) and iHeps (Day 16, right). (c) Morphology of untransduced MEFs cultured in MEF medium (left) and iHep medium (middle) and iHeps (right) in iHep medium for 16 days. Scale bar = 100 μm. MEFs: mouse embryonic fibroblasts [Color figure can be viewed at wileyonlinelibrary.com]

Previous studies showed that forced expression of a set of transcription factors could reprogram MEFs into hepatocyte lineage (Huang et al., 2011; Sekiya & Suzuki, 2011). This system however was based on separate expression vectors, which led to uncertain transduction of the individual genes in cells, leading to a heterogenous population. We questioned whether the all‐in‐one polycistronic expression system could overcome this issue. MEFs were transduced with the all‐in‐one lentivirus and fluorescence was observed after 2 days, which indicates the successful transduction and expression of exogenous genes (data not shown). Two days after transduction we changed the MEF culture medium to iHep culture medium (Figure 2a; medium components based on Sekiya and Suzuki (2011)). After 2 weeks, we observed that almost 90% of the cells were dTomato positive (Figure 2b). Besides, the cell morphology changed from elongated to polygonal shape (Figure 2c), which is a typical phenotype of hepatocytes. We designated these cells induced hepatocytes or iHeps.

**Figure 3:**
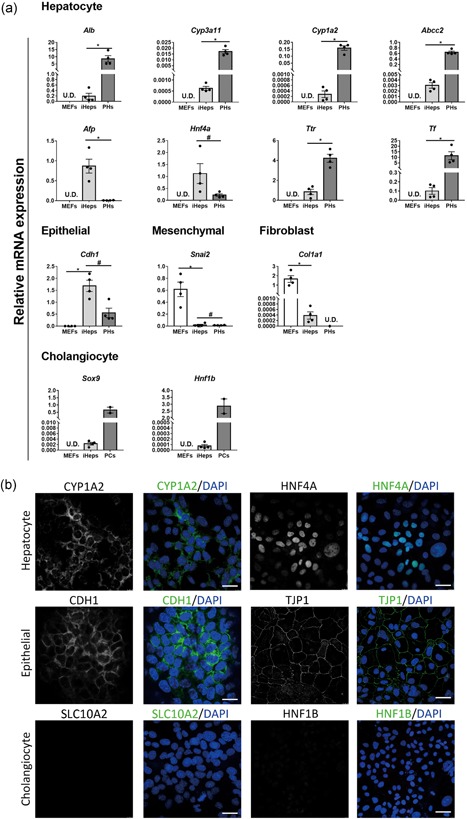
Characterization of iHeps. (a) Gene expression analysis for MEFs, iHeps, primary mouse hepatocytes (PHs), and primary cholangiocytes. Data are shown as mean ± *SEM* of four independent samples for each group. The asterisk represents statistical significance. *p < 0.05. The hash represents nonsignificance (two‐tailed Mann–Whitney *U* test). Hepatocyte markers, albumin (*Alb*), cytochrome P450 3a11 (*Cyp3a11*), cytochrome P450 1a2 (*Cyp1a2*), and ATP binding cassette subfamily C member 2 (*Abcc2*); epithelial marker, caderin1 (*Cdh1*); mesenchymal marker, snail family transcriptional repressor 2 (*Snai2*); fibroblast marker, collagen type I alpha 1 chain (*Col1a1*); cholangiocyte/hepatic progenitor markers, SRY‐box 9 (*Sox9*) and HNF1 homeobox B (*Hnf1b*). (b) Immunofluorescence analysis for iHeps. Hepatocyte markers, CYP1A2 and hepatocyte nuclear factor 4 alpha (HNF4A); epithelial markers, CDH1 and tight junction protein 1 (TJP1); cholangiocyte markers, solute carrier family 10 member 2 (SLC10A2) and HNF1B. Nuclear staining with 4′,6‐diamidino‐2‐phenylindole dihydrochloride (DAPI) for all conditions. Scale bar = 30 μm [Color figure can be viewed at wileyonlinelibrary.com]

Gene expression analysis demonstrated little expression of mesenchymal marker snail family transcriptional repressor 2 and fibroblast marker collagen type I alpha 1 chain in iHeps (Figure 3a). In contrast, epithelial marker cadherin 1 (*Cdh1*, also known as E‐cadherin) was highly expressed in iHeps (Figure 3a), which indicates the mesenchymal‐epithelial transition of cell fate. Furthermore, iHeps displayed increased expression of the following hepatocyte markers: *Alb*, cytochrome P450 family 1 subfamily A member 2 (*Cyp1a2*), cytochrome P450 family 3 subfamily A member 11 (*Cyp3a11*), ATP binding cassette subfamily C member 2 (*Abcc2*), alpha fetoprotein (*Afp*), hepatocyte nuclear factor 4 alpha, transthyretin, and transferrin (Figure 3a). The cholangiocyte or hepatic progenitor markers SRY‐box 9 (*Sox9*) and HNF1 homeobox B (*Hnf1b*) were expressed but at significantly lower levels compared with organoid‐derived cholangiocytes generated by previous protocol (unpublished data). Immunofluorescence analysis confirmed the presence of hepatocyte markers (CYP1A2 and HNF4A) and epithelial markers (CDH1 and tight junction protein 1 [TJP1]), and the absence of cholangiocyte markers (solute carrier family 10 member 2 [SLC10A2] and HNF1B; Figure 3b). Together, these results showed that direct reprogramming, through an all‐in‐one polycistronic expression system, can induce MEFs to iHeps without inducing differentiation into the cholangiocyte lineage.

### 3.3 Functional validation of iHeps

**Figure 4:**
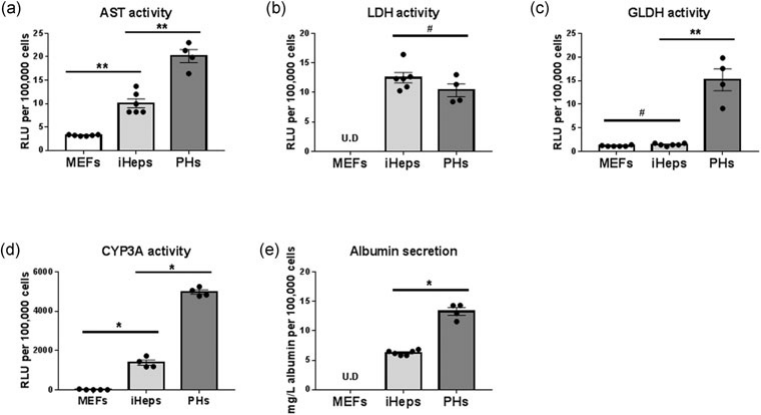
Functional validation of iHeps. Relative activity of hepatocyte‐specific enzymes (a–c), Cyp3a activity (d), and albumin secretion (e) in MEFs, iHeps, and primary hepatocytes. Results were normalized with cell number. Normalized data are shown as mean ± *SEM* of four independent experiments for primary hepatocytes and six independent experiments for MEFs and iHeps (P17). The asterisk represents statistical significance. *p < 0.05. *p < 0.01. The hash represents nonsignificance (two‐tailed Mann–Whitney *U* test). AST: aspartate aminotransferase; LDH: lactate dehydrogenase; GLDH: glutamate dehydrogenase; MEFs: mouse embryonic fibroblasts; *SEM*: standard error of the mean

Next, we investigated whether the iHeps generated by the all‐in‐one polycistronic expression system functionally resembled primary hepatocytes. AST, LDH, and LDH are intracellular enzymes catalyzing important metabolism functions, and they are specifically expressed in hepatocytes in the liver (Gowda et al., 2009; Jaeschke & McGill, 2013; Kotoh et al., 2011). We collected lysate from MEFs, iHeps, and primary hepatocytes, and measured their enzyme activities. Figure 4 shows that the activities of AST and LDH in iHeps were upregulated (Figure 4a,b), but GLDH activity remained at similar levels compared with MEFs (Figure 4c). LDH levels were similar to those observed in primary hepatocytes. Notably, GLDH is predominantly located in the centrilobular (Zone 3) region of the liver, whereas AST is more homogenously distributed (Dancygier, 2010), indicating that iHeps might represent hepatocytes with noncentrilobular origin. Besides, GGT and ALP, which are related to biliary tracts, were undetectable in MEFs, iHeps, and primary hepatocytes (data not shown), confirming the nonbiliary fate of iHeps.

The cytochrome P450 enzymes (CYP) are essential in drug metabolism, in particular CYP3A4 (mouse homologous isoforms are CYP3A11, CYP3A16, CYP3A41A, CYP3A41B, and CYP3A44; Cui, Renaud, & Klaassen, 2012). In iHeps, we observed that the CYP3A activity was induced to almost one third of the levels compared with primary hepatocytes (Figure 4d). Another key function of hepatocytes is producing serum proteins; therefore, we examined the *Alb* concentration in culture medium of iHeps. As Figure 4e shows, *Alb* was detectable in iHep culture medium reaching about half the concentration in primary hepatocyte culture medium.

### 3.4 Repopulation of DLD by iHeps

**Figure 5:**
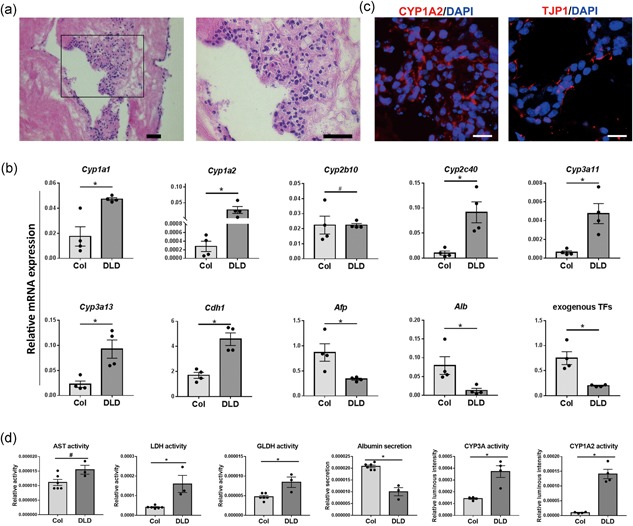
Repopulation of decellularized liver disks by iHeps. (a) Hematoxylin and eosin (H&E) staining showing the repopulation of decellularized liver disks by iHeps after 1 week. Scale bar = 50 μm. (b) Immunofluorescence analysis for iHeps cultured on decellularized liver disks (DLD). Scale bar = 20 μm. (c) Gene expression analysis for iHeps cultured on collagen (type I) coated plates (Col) and DLD. (d) Relative activity of hepatocyte‐specific enzymes (AST, LDH, GLDH), CYP3A and CYP1A2 activity and albumin secretion in iHeps cultured on Col and DLD. All results were normalized with cell input with Alamar blue. Data are shown as mean ± *SEM* of six independent experiments for collagen 2D cultured iHeps for AST, LDH, GLDH and albumin tests, three for DLD cultured iHeps for AST, LDH, GLDH and albumin tests, four for both 2D and DLD cultured iHeps for CYP3A and CYP1A2 tests. The asterisk represents statistical significance. *p < 0.05. The hash represents nonsignificance (two‐tailed Mann–Whitney *U* test) [Color figure can be viewed at wileyonlinelibrary.com]

Previously we and others have reported that hepatic stem cells and primary hepatocytes could repopulate decellularized liver tissue (Baptista et al., 2011; Soto‐Gutierrez et al., 2011; Vyas et al., 2018; Wang et al., 2011). We questioned whether iHeps also possessed the repopulation capacity. When iHeps were seeded on rat DLD, we found they survived and could repopulate DLD within 1 week (Figure 5a). In contrast, nonreprogrammed MEFs seeded on DLD did not survive (data not shown).

Because the ECM in DLD represents a natural habitat for hepatocytes, we determined whether DLD could further enhance the maturation of iHeps. Gene expression analysis demonstrates that cytochromes P450 including *Cyp1a1*, *Cyp1a2*, *Cyp2c40*, *Cyp3a11*, and *Cyp3a13*, and epithelial marker *Cdh1*, were dramatically upregulated, whereas the early hepatocyte marker *Afp* was downregulated (Figure 5b). Interestingly, we observed that *Alb* and exogenous FHG were significantly downregulated (Figure 5b,d). Immunofluorescence analysis demonstrates the expression of CYP1A2 and TJP1 (Figure 5c). Finally, iHeps cultured with DLD exhibited the increased enzyme activity of LDH, GLDH, CYP3A, and CYP1A2 (Figure 5d), whereas GGT and ALP activity were undetectable (data not shown). Together, these results indicate that iHeps reached a high level of maturation. Although *Alb* expression was downregulated, the iHep‐on‐decellularized‐liver‐tissue system still represents an excellent model for phase I drug metabolism studies.

## 4 DISCUSSION

Direct reprogramming of fibroblasts into hepatocytes is time‐saving and requires less effort due to the bypass of an intermediate stage of pluripotency; hence, it represents an optimal way to quickly generate a large population of cells. The heterogeneity of iHeps and their lack of liver function may be due to the uncontrollable expression proportion of hepatocyte‐generating factors FHG separately by means of monocistronic vectors. To overcome this issue, an all‐in‐one polycistronic expression system is needed. The classical and widely used way to construct bicistronic or polycistronic vectors is to insert an IRES between genes. Because of its large molecular size and the difference in expression levels between genes before and after IRES (the gene before IRES generally has disproportional higher expression), we used the 2A peptide sequence insert in this study. This 2A peptide sequence is usually 50–60 bp compared with around 580 bp of IRES, which enables the construction of vectors containing four or even more encoding regions. This is especially important for a lenti‐ and retroviral expression system, of which the size of packaged sequence is limited (Kumar, Keller, Makalou, & Sutton, 2001). Besides, genes before and after the 2A peptides are separated from each other during posttranslational modification by a highly efficient cleavage event; therefore, functional proteins can be obtained. The all‐in‐one polycistronic expression system used in this study induced high expression of all genes at messenger RNA and protein level, which allowed the generation of iHeps.

During in vitro culture at normal conditions MEFs become senescent or reach their Hayflick limit, after 5–7 passages (Amand, Hanover, & Shiloach, 2016). To our surprise, the transduced MEFs or iHeps (transduction performed at passage 4) continued to grow for at least 30 passages over 2 months (longer term to be determined). This growth advantage of iHeps provided us an easy way to select the positive cells over untransduced MEFs. We observed that almost 90% cells were dTomato positive after 2 weeks and their morphology was identical after two passages (Figure 2). With further (sub)culture, this percentage kept increasing and a homogenous population was generated without the need of fluorescence‐activated cell sorting. As hepatocytes do not proliferate in vitro, the continued proliferation of iHeps indicates that these cells are not fully mature, which is also reflected by other results, such as high expression of the early hepatocyte marker *Afp* and relatively low expression of the late hepatocyte markers *Cyp3a11* and *Cyp1a2* (Figure 3). Nevertheless, this is a clear advantage when large numbers of cells are needed for tissue engineering or cell therapy approaches.

To improve the maturation of iHeps, we introduced iHeps into decellularized liver tissue. This natural‐based ECM, mainly containing collagens, laminin, and fibronectin, does not only provide mechanical and structural support for cells, but also serves as the reservoir for growth factors like HGF and bFGF (Soto‐Gutierrez et al., 2011). After seeding on DLD, we observed that the exogenous expression of FHG was downregulated. This explains that *Alb*, a direct downstream target of these three transcription factors (Cirillo et al., 2002; Kajiyama, Tian, & Locker, 2006; Lichtsteiner & Schibler, 1989), also showed lower expression. Although the precise mechanism behind the quenching of exogenous gene expression is unknown, it is a relatively common phenomenon during cell fate reprogramming (Huang et al., 2011; Takahashi & Yamanaka, 2006) and indicates the dominance of the microenvironment. Having said this, the drug metabolism potential (*Cyp3a11* and *Cyp1a2*) of iHeps was greatly enhanced and reached levels that were close to primary hepatocytes. The generation of the DLD is relatively easy and highly reproducible (Wang et al., 2017). The combined robust differentiation capability together with the disks results in a liver model that is more representative of the native liver tissue and has comparable hepatic features. These results indicate that the iHep‐on‐decellularized‐liver‐tissue system may represent an excellent model for phase I drug metabolism studies.

There are a few limitations in this study. The primary mouse hepatocytes were isolated by a two‐step collagen perfusion method (Walker, Jackson, Taylor, Jones, & Forrester, 2010). Although the majority of the isolated population were hepatocytes, the presence of some other unwanted cell types (e.g., hepatic stellate cells, cholangiocytes, etc.) is inevitable. Therefore, we used a commercial media designated for hepatocytes culture (HepatoZYME; Gibco) to select out other cell types. Because in vitro hepatocytes cultures could de‐differentiate rapidly (Chen, Wong, Sjeklocha, Steer, & Sahin, 2012), our culture of 2 days before measurement could have influenced the results. This limitation could be overcome by utilizing freshly isolated pure hepatocytes (e.g., by sorting ASGPR^+^ cells; Severgnini et al., 2012) as the most optimal reference cell type. Second, the decellularized liver tissue used in this study was derived from rats as no decellularized mouse material was available. The species incompatibility of mouse cells and rat ECM might limit the full maturation potential of iHeps.

## 5 CONCLUSION

Here we reported an easy and efficient way to generate expandable iHeps. Applying decellularized liver tissue to the in vitro culture resulted in iHeps with enhanced hepatic features. Our finding demonstrates that hepatic ECM can induce important functions of iHeps derived from somatic cells by direct reprogramming. Future research may focus on introducing supporting cell types (e.g., hepatic stellate cells, mesenchymal stromal cells, endothelial cells, etc.) to the current system to generate an even more physiological microenvironment for hepatocytes.

## CONFLICTS OF INTEREST

The authors have declared no conflicts of interest.

## AUTHOR CONTRIBUTIONS

C. C. contributed to collection and/or assembly of data, data analysis and interpretation, and manuscript writing. I. P.‐P. provided study material. P. M. B. conceptualized and designed the study, and analyzed and interpreted the data. P.S., L. A. O., and M. E. V. collected and/or assembled the data. L.C.P. and N.G. analyzed and interpreted the data and gave the final approval of the manuscript. B.S. conceptualized and designed the study, analyzed and interpreted the data, and gave the final approval of the manuscript.

## Supporting information

Supporting information

## References

[bit26784-bib-0001] Amand, M. M. , Hanover, J. A. , & Shiloach, J. (2016). Journal of Biological Methods, 3(2), 41 https://doi.org/http://.org/10.14440/jbm.2016.110. 10.14440/jbm.2016.110 PMC6706133 31453208

[bit26784-bib-0002] Baptista, P. M. , Siddiqui, M. M. , Lozier, G. , Rodriguez, S. R. , Atala, A. , & Soker, S. (2011). The use of whole organ decellularization for the generation of a vascularized liver organoid. Hepatology, 53(2), 604–617. https://doi.org/http://.org/10.1002/hep.24067. 21274881 10.1002/hep.24067

[bit26784-bib-0003] Baum, C. , Hegewisch‐Becker, S. , Eckert, H. G. , Stocking, C. , & Ostertag, W. (1995). Novel retroviral vectors for efficient expression of the multidrug resistance (mdr‐1) gene in early hematopoietic cells. Journal of Virology, 69(12), 7541–7547. Retrieved from. http://www.ncbi.nlm.nih.gov/pubmed/7494260%5Cnhttp://www.pubmedcentral.nih.gov/articlerender.fcgi?artid. 7494260 10.1128/jvi.69.12.7541-7547.1995 PMC189692

[bit26784-bib-0004] Brangel, P. , & Undine, S. (2016). Annual Report 2016 Eurotransplant International Foundation. Eurotransplant International Foundation.

[bit26784-bib-0005] Chen, Y. , Wong, P. P. , Sjeklocha, L. , Steer, C. J. , & Sahin, M. B. (2012). Mature hepatocytes exhibit unexpected plasticity by direct dedifferentiation into liver progenitor cells in culture. Hepatology, 55(2), 563–574. https://doi.org/http://.org/10.1002/hep.24712. 21953633 10.1002/hep.24712 PMC3268884

[bit26784-bib-0006] Cirillo, L. A. , Lin, F. R. , Cuesta, I. , Friedman, D. , Jarnik, M. , & Zaret, K. S. (2002). Opening of compacted chromatin by early developmental transcription factors HNF3 (FoxA) and GATA‐4. Molecular Cell, 9(2), 279–289. https://doi.org/http://.org/10.1016/S1097-2765(02)00459-8. 11864602 10.1016/s1097-2765(02)00459-8

[bit26784-bib-0007] Cui, J. Y. , Renaud, H. J. , & Klaassen, C. D. (2012). Ontogeny of novel cytochrome P450 gene isoforms during postnatal liver maturation in mice. Drug Metabolism and Disposition, 40(6), 1226–1237. https://doi.org/http://.org/10.1124/dmd.111.042697. 22446519 10.1124/dmd.111.042697 PMC3362787

[bit26784-bib-0008] Dancygier, H. (2010). Clinical Hepatology, Berlin, Germany: Springer https://doi.org/http://.org/10.1007/978-3-540-93842-2.

[bit26784-bib-0009] Du, Y. , Wang, J. , Jia, J. , Song, N. , Xiang, C. , Xu, J. , … Deng, H. (2014). Human hepatocytes with drug metabolic function induced from fibroblasts by lineage reprogramming. Cell Stem Cell, 14(3), 394–403. https://doi.org/http://.org/10.1016/j.stem.2014.01.008. 24582926 10.1016/j.stem.2014.01.008

[bit26784-bib-0010] Gowda, S. , Desai, P. B. , Hull, V. V. , Math, A. A. , Vernekar, S. N. , & Kulkarni, S. S. (2009). A review on laboratory liver function tests. The Pan African Medical Journal, 3(November), 17 https://doi.org/http://.org/10.11604/pamj.2009.3.17.125. 21532726 PMC2984286

[bit26784-bib-0011] Huang, P. , He, Z. , Ji, S. , Sun, H. , Xiang, D. , Liu, C. , … Hui, L. (2011). Induction of functional hepatocyte‐like cells from mouse fibroblasts by defined factors. Nature, 475(7356), 386–389. https://doi.org/http://.org/10.1038/nature10116. 21562492 10.1038/nature10116

[bit26784-bib-0012] Huang, P. , Zhang, L. , Gao, Y. , He, Z. , Yao, D. , Wu, Z. , … Hui, L. (2014). Direct reprogramming of human fibroblasts to functional and expandable hepatocytes. Cell Stem Cell, 14(3), 370–384. https://doi.org/http://.org/10.1016/j.stem.2014.01.003. 24582927 10.1016/j.stem.2014.01.003

[bit26784-bib-0013] Jaeschke, H. , & McGill, M. R. (2013). Serum glutamate dehydrogenase‐biomarker for liver cell death or mitochondrial dysfunction? Toxicological Sciences, 134(1), 221–222. https://doi.org/http://.org/10.1093/toxsci/kft087. 23568080 10.1093/toxsci/kft087

[bit26784-bib-0014] Kajiyama, Y. , Tian, J. , & Locker, J. (2006). Characterization of distant enhancers and promoters in the albumin‐α‐fetoprotein locus during active and silenced expression. Journal of Biological Chemistry, 281(40), 30122–30131. https://doi.org/http://.org/10.1074/jbc.M603491200. 16893898 10.1074/jbc.M603491200

[bit26784-bib-0015] Kelaini, S. , Cochrane, A. , & Margariti, A. (2014). Direct reprogramming of adult cells: Avoiding the pluripotent state. Stem Cells and Cloning: Advances and Applications, 7(1), 19–29. https://doi.org/http://.org/10.2147/SCCAA.S38006. 24627642 10.2147/SCCAA.S38006 PMC3931695

[bit26784-bib-0016] Kim, J. H. , Lee, S. R. , Li, L. H. , Park, H. J. , Park, J. H. , Lee, K. Y. , & Choi, S. Y. (2011). High cleavage efficiency of a 2A peptide derived from porcine teschovirus‐1 in human cell lines, zebrafish and mice. PLoS One, 6(4), 1–8. https://doi.org/http://.org/10.1371/journal.pone.0018556. 10.1371/journal.pone.0018556 PMC3084703 21602908

[bit26784-bib-0017] Kotoh, K. , Kato, M. , Kohjima, M. , Tanaka, M. , Miyazaki, M. , Nakamura, K. , … TAKAYANAGI, R. (2011). Lactate dehydrogenase production in hepatocytes is increased at an early stage of acute liver failure. Experimental and Therapeutic Medicine, 2(2), 195–199. https://doi.org/http://.org/10.3892/etm.2011.197. 22977488 10.3892/etm.2011.197 PMC3440653

[bit26784-bib-0018] Kumar, M. , Keller, B. , Makalou, N. , & Sutton, R. E. (2001). Systematic determination of the packaging limit of lentiviral vectors. Human Gene Therapy, 12(15), 1893–1905. https://doi.org/http://.org/10.1089/104303401753153947. 11589831 10.1089/104303401753153947

[bit26784-bib-0019] LeCluyse, E. L. , Bullock, P. L. , & Parkinson, A. (1996). Strategies for restoration and maintenance of normal hepatic structure and function in long‐term cultures of rat hepatocytes. Advanced Drug Delivery Reviews, 22(1–2), 133–186. https://doi.org/http://.org/10.1016/S0169-409X(96)00418-8.

[bit26784-bib-0020] Lichtsteiner, S. , & Schibler, U. (1989). A glycosylated liver‐specific transcription factor stimulates transcription of the albumin gene. Cell, 57(7), 1179–1187. 10.1016/0092-8674(89)90055-X. 2736625

[bit26784-bib-0021] Nantasanti, S. , Spee, B. , Kruitwagen, H. S. , Chen, C. , Geijsen, N. , Oosterhoff, L. A. , … Schotanus, B. A. (2015). Disease modeling and gene therapy of copper storage disease in canine hepatic organoids. Stem Cell Reports, 5(5), 895–907. https://doi.org/http://.org/10.1016/j.stemcr.2015.09.002. 26455412 10.1016/j.stemcr.2015.09.002 PMC4649105

[bit26784-bib-0022] Schambach, A. , Bohne, J. , Baum, C. , Hermann, F. G. , Egerer, L. , von Laer, D. , & Giroglou, T. (2006). Woodchuck hepatitis virus post‐transcriptional regulatory element deleted from X protein and promoter sequences enhances retroviral vector titer and expression. Gene Therapy, 13(7), 641–645. https://doi.org/http://.org/10.1038/sj.gt.3302698. 16355114 10.1038/sj.gt.3302698

[bit26784-bib-0023] Schwartz, R. E. , Fleming, H. E. , Khetani, S. R. , & Bhatia, S. N. (2014). Pluripotent stem cell‐derived hepatocyte‐like cells. Biotechnology Advances, 32(2), 504–513. https://doi.org/http://.org/10.1016/j.biotechadv.2014.01.003. 24440487 10.1016/j.biotechadv.2014.01.003 PMC4043206

[bit26784-bib-0024] Sekiya, S. , & Suzuki, A. (2011). Direct conversion of mouse fibroblasts to hepatocyte‐like cells by defined factors. Nature, 475(7356), 390–393. https://doi.org/http://.org/10.1038/nature10263. 21716291 10.1038/nature10263

[bit26784-bib-0025] Severgnini, M. , Sherman, J. , Sehgal, A. , Jayaprakash, N. K. , Aubin, J. , Wang, G. , … Fitzgerald, K. (2012). A rapid two‐step method for isolation of functional primary mouse hepatocytes: Cell characterization and asialoglycoprotein receptor based assay development. Cytotechnology, 64(2), 187–195. 10.1007/s10616-011-9407-0. 22105762 PMC3279583

[bit26784-bib-0026] Soto‐Gutierrez, A. , Zhang, L. , Medberry, C. , Fukumitsu, K. , Faulk, D. , Jiang, H. , … Badylak, S. F. (2011). A whole‐organ regenerative medicine approach for liver replacement. Tissue Engineering Part C: Methods, 17(6), 677–686. 10.1089/ten.tec.2010.0698. 21375407 PMC3103054

[bit26784-bib-0027] Takahashi, K. , & Yamanaka, S. (2006). Induction of pluripotent stem cells from mouse embryonic and adult fibroblast cultures by defined factors. Cell, 126(4), 663–676. https://doi.org/http://.org/10.1016/j.cell.2006.07.024. 16904174 10.1016/j.cell.2006.07.024

[bit26784-bib-0028] van Steenbeek, F. G. , Spee, B. , Penning, L. C. , Kummeling, A. , van Gils, I. H. M. , Grinwis, G. C. M. , … Leegwater, P. A. J. (2013). Altered Subcellular Localization of Heat Shock Protein 90 Is Associated with Impaired Expression of the Aryl Hydrocarbon Receptor Pathway in Dogs. PLoS One, 8(3), e57973 https://doi.org/http://.org/10.1371/journal.pone.0057973. 23472125 10.1371/journal.pone.0057973 PMC3589449

[bit26784-bib-0029] Vyas, D. , Baptista, P. M. , Brovold, M. , Moran, E. , Gaston, B. , Booth, C. , & Soker, S. (2018). Self‐assembled liver organoids recapitulate hepatobiliary organogenesis in vitro. Hepatology, 1, 1–36. https://doi.org/http://.org/10.1002/hep.29483. 10.1002/hep.29483 PMC5825235 28834615

[bit26784-bib-0030] Walker, J. M. , Jackson, M. , Taylor, a H. , Jones, E. a , & Forrester, L. M. (2010). Mouse Cell Culture. Methods in Molecular Biology, 633, https://doi.org/http://.org/10.1007/978-1-59745-019-5. 10.1007/978-1-59745-019-5_1 20204616

[bit26784-bib-0031] Wang, Y. , Nicolas, C. T. , Chen, H. S. , Ross, J. J. , De Lorenzo, S. B. , & Nyberg, S. L. (2017). Recent advances in decellularization and recellularization for tissue‐engineered liver grafts. Cells, Tissues, Organs, 204(3–4), 125–136. https://doi.org/http://.org/10.1159/000479597. 28972946 10.1159/000479597

[bit26784-bib-0032] Wang, Y. , Cui, C. B. , Yamauchi, M. , Miguez, P. , Roach, M. , Malavarca, R. , … Reid, L. M. (2011). Lineage restriction of human hepatic stem cells to mature fates is made efficient by tissue‐specific biomatrix scaffolds. Hepatology, 53(1), 293–305. https://doi.org/http://.org/10.1002/hep.24012. 21254177 10.1002/hep.24012

[bit26784-bib-0033] Warlich, E. , Kuehle, J. , Cantz, T. , Brugman, M. H. , Maetzig, T. , Galla, M. , … Schambach, A. (2011). Lentiviral vector design and imaging approaches to visualize the early stages of cellular reprogramming. Molecular Therapy: The Journal of the American Society of Gene Therapy, 19(4), 782–789. https://doi.org/http://.org/10.1038/mt.2010.314. 21285961 10.1038/mt.2010.314 PMC3070104

[bit26784-bib-0034] Wu, X.‐B. , & Tao, R. (2012). Hepatocyte differentiation of mesenchymal stem cells. Hepatobiliary & Pancreatic Diseases International, 11(4), 360–371. https://doi.org/http://.org/10.1016/S1499-3872(12)60193-3. 22893462 10.1016/s1499-3872(12)60193-3

