# Supplementary material for: Hepatocyte‐like cells generated by direct reprogramming from murine somatic cells can repopulate decellularized livers
Source: Biotechnol Bioeng. 2018 Sep 17;115(11):2807–16. doi: 10.1002/bit.26784 (PMC6221165; doi:10.1002/bit.26784)
Supplement: Supplementary file 1 — Supporting information [file BIT-115-2807-s001.docx]

**Supplementary figures**


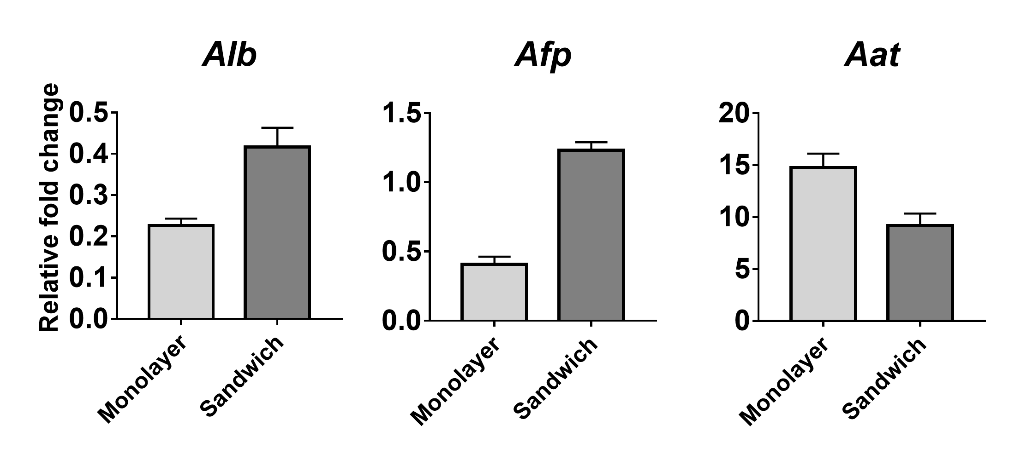


Figure S1. Comparison between monolayer and sandwich cultured iHeps. Gene expression analysis for iHeps cultured on monolayer and sandwich with collagen type I. Data are shown as mean ± SEM of two independent samples for each group.


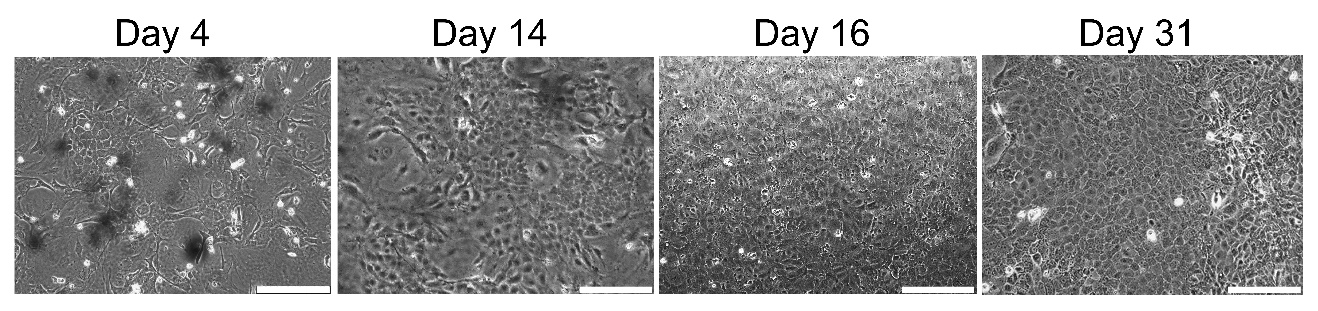


Figure S2. Morphological change during the generation of iHeps. MEFs were transduced on Day 0, photos were taken on Day 4, Day 14 (before the second passaging), Day 16 and Day 31. Scale bar = 200 μm.

**
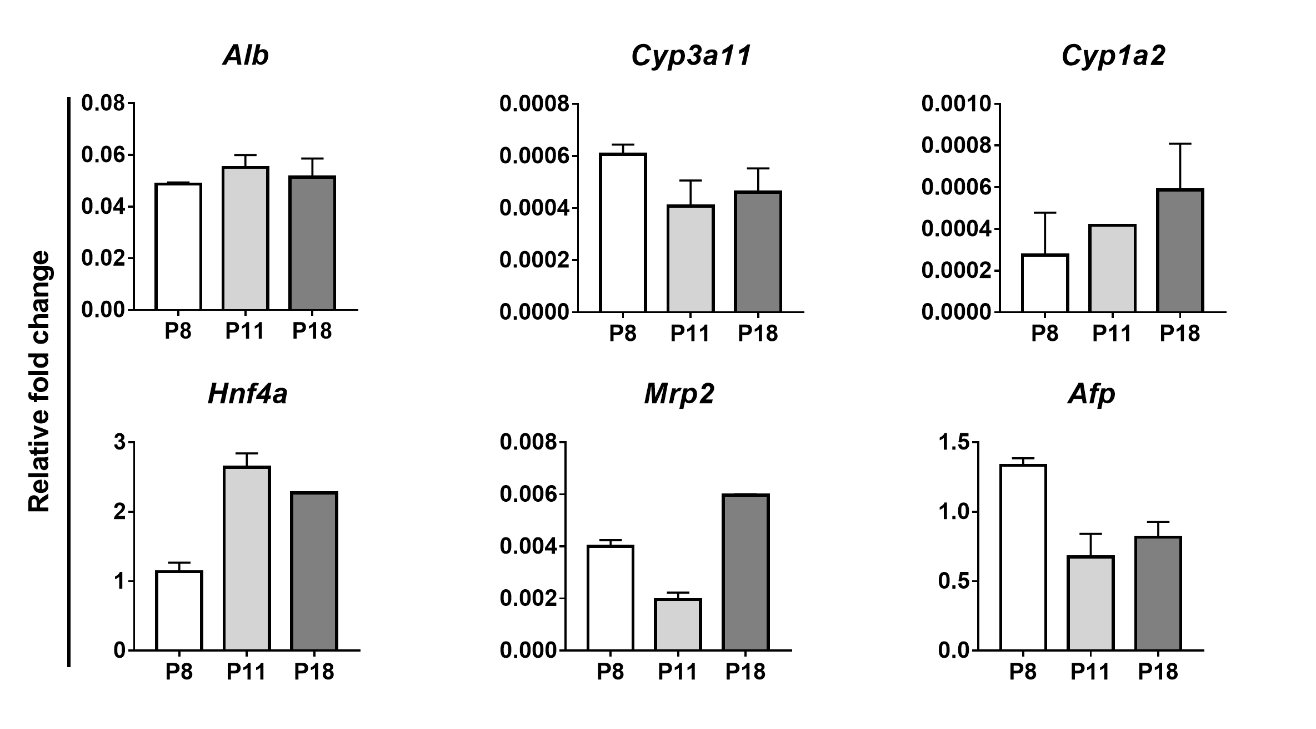
**

Figure S3. Comparison among iHeps at different passages. RNA samples were collected from iHeps at P8, P11 and P18. Data are shown as mean ± SEM of two independent samples for each group.


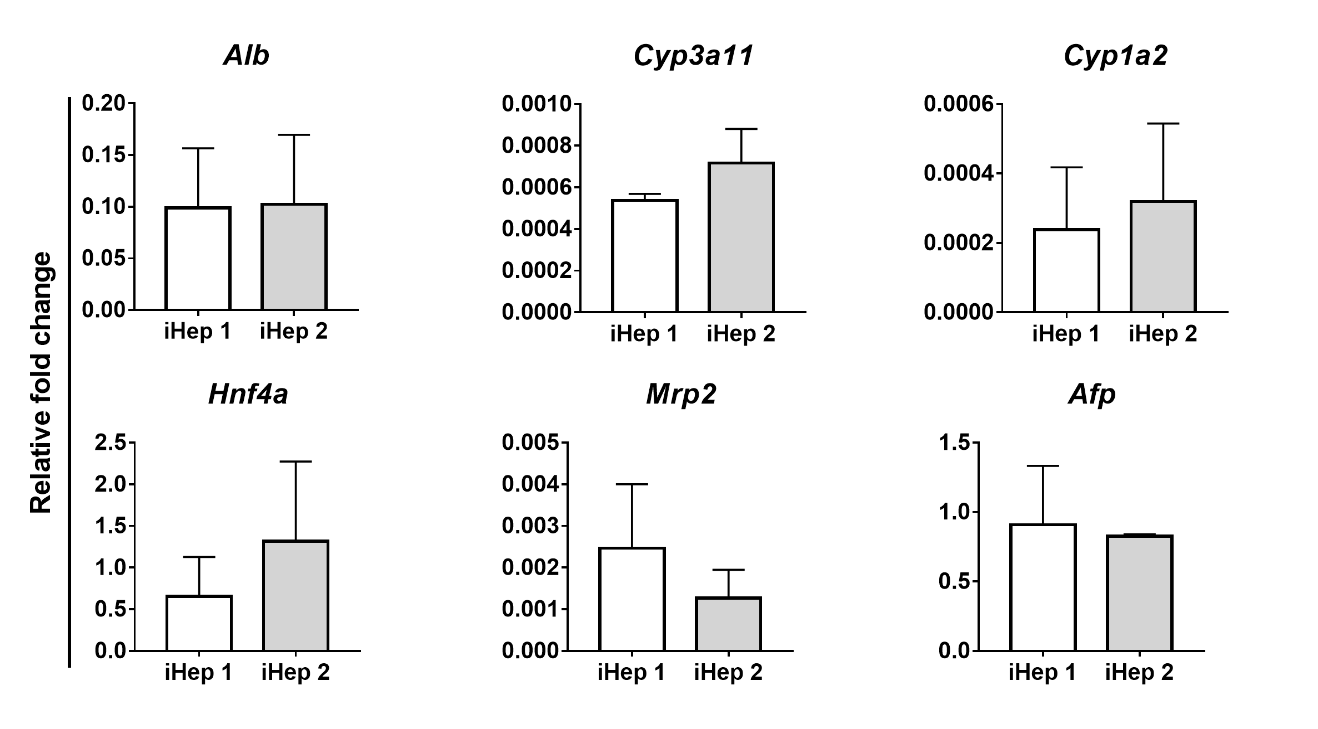


Figure S4. Comparison between iHeps generated from different donors. RNA samples were collected from iHep 1 and 2. Data are shown as mean ± SEM of two independent samples for each group.

**Supplemental tables**

**Supplemental Table 1. List of primers used in RT-qPCR analysis**

| **Gene** | **Direction** | **Sequence (5’ → 3’)** |
| --- | --- | --- |
| *Alb* | Forward | GCAACACAAAGATGACAACC |
|  | Reverse | CTTCATGCAAATAGTGTCCCA |
| *Hnf1b* | Forward | ACAATCCCCAGCAATCTCAGAA |
|  | Reverse | GCTGCTAGCCACACTGTTAATGA |
| *Hprt* | Forward | GTGATTAGCGATGATGAACCA |
|  | Reverse | CAAGTCTTTCAGTCCTGTCCA |
| *Sox9* | Forward | CCCGATTACAAGTACCAGCC |
|  | Reverse | CCCTGAGATTGCCCAGAGTG |
| *Cyp3a11* | Forward | AAACTCAAGGAGATGTTCCC |
|  | Reverse | TTCACTCCAAATGATGTGCT |
| *Abcc2* | Forward | GATAGCCTCATTCAGACGAC |
|  | Reverse | ACCATTATCTTGTCACTGTCCA |
| *Cyp1a2* | Forward | ACCATGATGAGAAGCAGTGGA |
|  | Reverse | CGAAGAGCATCACCTTCTCG |
| *Col1a1* | Forward | CTGGTTCTCCTGGTTCTCCT |
|  | Reverse | CGTTGAGTCCGTCTTTGCC |
| *Snai2* | Forward | CTCCAAGAAGCCCAACTACAG |
|  | Reverse | TGCCGACGATGTCCATACAG |
| *Afp* | Forward | TCGTATTCCAACAGGAGG |
|  | Reverse | AGGCTTTTGCTTCACCAG |
| *Ttr* | Forward | TAGAACTGGACACCAAATCGT |
|  | Reverse | CTGTAGGAGTATGGGCTGAG |
| *Tf* | Forward | CTCTTGAGAAAGCTGTGTCC |
|  | Reverse | AAAGAATGGTTGAGTGGAGG |
| *Cdh1* | Forward | GAGAGAGACTGGAGTGCCACC |
|  | Reverse | CTGTGTACCCTCACCATCGG |
| *Cyp1a1* | Forward | ACGAGAATGCCAATGTCCAG |
|  | Reverse | CCAATCACTGTGTCTAGTTCCT |
| *Cyp3a13* | Forward | AGGCTCAAGGAGATGTTCCC |
|  | Reverse | AGATGTCTTTCATGCTGGTGG |
| *Cyp2b10* | Forward | GGAACAGACACCATAAGGGA |
|  | Reverse | GCAAAGATCACACCATATTCCT |
| *Cyp2c40* | Forward | TGGACTATACCATTGAACACCT |
|  | Reverse | CTGGACTTTAGCTGTGATGTG |

**Supplemental Table 2. List of antibodies used in immunofluorescence analysis**

| **Antibody** | **Species** | **Supplier** | **Cat. No.** | **Dilution** |
| --- | --- | --- | --- | --- |
| HNF1B | Rabbit | Sigma | HPA002083 | 1:400 |
| TJP1 | Rabbit | Invitrogen | 40-2300 | 1:250 |
| CDH1 | Mouse | BD Biosciences | 610181 | 1:100 |
| CYP1A2 | Rabbit | Abcam | Ab77795 | 1:600 |
| HNF4A | Rabbit | Santa Cruz | sc-8987 | 1:500 |
| SLC10A2 | Rabbit | Bioss | bs-4189R | 1:200 |
